# A Case-control Study in Hiroshima and Nagasaki Examining Non-radiation Risk Factors for Thyroid Cancer

**DOI:** 10.2188/jea.17.76

**Published:** 2007-06-02

**Authors:** Jun Nagano, Kiyohiko Mabuchi, Yasuhiko Yoshimoto, Yuzo Hayashi, Nobuo Tsuda, Charles Land, Kazunori Kodama

**Affiliations:** 1 Department of Epidemiology, Radiation Effects Research Foundation.; 2 Institute of Health Science, Kyushu University.; 3 Radiation Epidemiology Branch, Division of Cancer Epidemiology and Genetics, National Cancer Institute.; 4 National Institute of Radiological Sciences; 5 Department of Pathology, Hiroshima City, Asa Hospital.; 6 Department of Pathology, Nagasaki Prefecture Medical Health Group Program.

**Keywords:** Thyroid Neoplasms, Smoking, Alcohol Drinking, Radiation, Case-Control Studies

## Abstract

**BACKGROUND:**

Because little is known about the etiology of thyroid cancer in Japan, we conducted a case-control study of thyroid cancer and lifestyle and other risk factors. The present report focuses on medical history, family history, smoking and alcohol drinking, and their interactions with radiation exposure.

**METHODS:**

Thyroid cancer cases reported to the Hiroshima and Nagasaki tumor registries during 1970-1986 were histologically reviewed by pathologists. For each of 362 cases with papillary or follicular adenocarcinoma diagnosed at <75 years of age, one control without cancer matched on city, sex, year of birth, and atomic-bomb radiation exposure was selected from the Life Span Study cohort or the offspring cohort. The cohort subjects were residents of Hiroshima and Nagasaki with or without atomic-bomb radiation exposure. Information on risk factors was obtained through a pre-structured interview carried out in 1986-1988.

**RESULTS:**

Analysis using conditional logistic regression showed history of goiter or thyroid nodule and family history of cancer to be significantly associated with an increased odds ratio for thyroid cancer. Smoking and alcohol drinking were significantly and independently associated with a reduced odds ratio. Interaction between smoking and alcohol drinking was not evident based on either an additive model or a multiplicative model. Radiation exposure did not significantly modify the associations between these factors and thyroid cancer risk.

**CONCLUSION:**

History of goiter/nodule and family history of cancer were risk factors for thyroid cancer. Smoking and alcohol drinking were independently associated with reduced risk. Self-reported retrospective information presents some limitations in interpretation of the data.

Cancer of the thyroid is relatively uncommon and represents less than 2-3 % of all incident cancers in many parts of the world.^1^^,^^2^ Papillary carcinoma is the most common histological type and accounts for 50-80% of thyroid cancer cases while follicular carcinomas make up 10-40%. Highly fatal anaplastic carcinoma and non-epithelial medullary carcinoma are much less frequent.^1^ Exposure to ionizing radiation is a well-established cause of thyroid cancer, especially non-medullary carcinomas.^1^^,^^3^ Recent evidence also implicates a history of goiter or thyroid nodules as a risk factor for thyroid cancer.^4^ Papillary carcinoma rates are higher in females than in males, especially at reproductive ages, suggesting a hormonal involvement.^1^ Ecological data suggest that papillary carcinomas tend to occur more frequently in areas of high iodine intake whereas follicular carcinomas occur more frequently in iodine-deficient areas.^5^ Several studies suggest a protective association of cigarette smoking, and of alcohol drinking less consistently, however.^6^

The incidence of thyroid cancer is relatively high in Japan,^2^ and is characterized by the predominance of papillary carcinomas.^7^ However, little is known about the etiology of thyroid cancer in Japan. In a hospital-based case-control study of female thyroid cancer, an increased risk was related to history of benign thyroid diseases and high parity while a decreased risk was related to Western style breakfast and coffee consumption.^8^ To our knowledge, no other Japanese study has investigated the association between lifestyle factors and thyroid cancer.

We conducted a case-control study of thyroid cancer in Hiroshima and Nagasaki to investigate a number of (potential) risk factors, including medical history, anthropometric factors, smoking and drinking habits, dietary habits, and female reproductive and menstrual factors, and to examine interaction between these factors and radiation exposure. Data on several specific risk factors from this study have been included in the international pooled analysis of 14 case-control studies of thyroid cancer.^4^^,^^6^ The pooled analyses, however, only used a part of the data in each study area, and presented a brief summary on the methods.^9^ We carried out analyses of the entire dataset including all risk factors queried. The present paper provides information on study design and data collection in detail, and provides results regarding past medical history, family history, smoking and alcohol drinking. Most of the present results were not included in the previous pooled analyses, i.e., past history of non-thyroidal diseases; family history of cancer; smoking and alcohol drinking considering mutual confounding effects and interaction between the two factors; and possible effect modification by ionizing radiation exposure from the atomic bombings.

## METHODS

### Subjects

We initially identified incident thyroid cancer cases born in 1910 or later and diagnosed during 1970-1986 from the tumor registries in Hiroshima and Nagasaki.^10^ These registries provide population-based coverage of incident cancers occurring among the residents of Hiroshima and Nagasaki. Two pathologists (Y.H. and N.T.) reviewed 645 thyroid cancer cases for whom tumor pathology slides were available, and confirmed thyroid cancer in 528 (82%). We excluded 16 who had died before interview and 74 who were found to be living outside the tumor registry catchment areas. Interviews were attempted with the remaining 438 subjects and completed with 386 (88%).

Because population-based rosters or other means by which to identify control subjects in the community were not available for the present study, we selected controls from members of the Life Span Study or offspring (F1) cohort who were residing in the Hiroshima and Nagasaki areas at the time of this study. The Life Span Study cohort of over 120,000 persons was set up to study the late effects of radiation among atomic-bomb survivors.^11^ This cohort, however, includes a large number of persons (more than 57,000) with little or negligible radiation exposure, selected randomly from the Hiroshima/Nagasaki residents who were distally exposed to the atomic bombs or who were not in either city at the time of the bombings. The F1 cohort consists of 76,814 persons born to atomic-bomb survivors or controls.^12^ The two cohorts are being followed up by the Radiation Effects Research Foundation (RERF), and further cohort details are described elsewhere.^11^^-^^13^

For each thyroid cancer case born before 1945, one control was selected from members of the Life Span Study cohort, which includes only persons born before the atomic bombings in 1945. Controls were alive at the time of the study, had no history of cancer and were matched by city of residence, sex, year of birth, and atomic-bomb radiation exposure. For cases in the Life Span Study and their controls, the matching on radiation exposure was based on DS86 estimated dose (see below); for a case with “unknown dose”, i.e., an exposed person for whom a dose estimate was not available, a control with unknown dose was selected. For cases born after 1945, controls were selected from members of the F1 cohort (born after 1945) by matching on city, sex and year of birth. Parental exposure to atomic bomb radiation was not considered in matching. Interviews were successfully completed for 87% of 522 controls.

The present analysis involved 362 papillary or follicular carcinoma case-control pairs. Excluded were cases who were 75 years old or over at the time of interview, and cases lacking an appropriate matched control. In addition, there were one anaplastic carcinoma case and two medullary carcinoma cases, and they were also excluded in the present analysis.

### Measures

Interviews were carried out between 1986 and 1988 by trained public health nurses who were not aware of the case-control status of the study subject. A pre-structured questionnaire was administered with 95% of the cases-control pairs while, for 5% of case-control pairs, surrogates (usually spouses) were interviewed. Questions were asked about histories regarding several specific thyroid condition, other diseases and radiotherapy to the head and neck region; family history of thyroid cancer and other cancer; anthropometric factors; lifestyle factors including smoking and alcohol drinking histories and dietary habits; and female reproductive and menstrual histories among women. Retrospective information was obtained up to the time of cancer diagnosis for cases, or up to the index time for controls. The index time for a control was when his/her counterpart case was diagnosed with thyroid cancer. Current smokers were those who regularly smoked cigarettes at the time of cancer diagnosis (or index time), and past smokers were those who had quit smoking before the time. As for alcohol drinking habits, consumption frequency at the time of cancer diagnosis (or index time) was queried for selected beverages, namely beer, sake, hard liquor, and whisky, with a pre-coded answer of “every day”, “almost every day”, “2-4 times per week”, “once per week”, “2-3 times per month”, “once per month”, “less than once per month”, and “none”. Persons drinking none of the beverages and those drinking any beverage every day were considered “never drinkers” and “daily drinkers”, respectively. Persons other than never and daily drinkers were regarded as “non-daily drinkers”.

For the Life Span Study subjects, individual radiation dose estimates were based on the dosimetry system DS86.^14^^,^^15^ This system provides estimates of individual gamma-ray and neutron shielded kerma and organ doses based on information about a survivor’s location and surrounding shielding conditions. We used the DS86 weighted thyroid dose, computed as gamma-ray dose plus ten times the neutron dose in units of sieverts (SV), to allow for the higher radiobiological effectiveness of neutrons compared to gamma-rays. By matching on radiation exposure, the main effect of radiation could not be assessed but a possible modification of other factors’ effects by radiation exposure could be examined (as described below).

### Statistical Analysis

The associations between exposure factors and thyroid cancer risk were examined by estimating odds ratios (ORs) with corresponding 95% confidence intervals (CIs), as well as by testing a dose-response relationship if necessary, using a conditional logistic regression model. When there was no subject in either cases or controls for a given factor, the Fisher’s exact test was applied. To examine whether the effect of a non-radiation factor is modified by atomic-bomb radiation exposure, case-control pairs were assigned an indicator variable for “exposed” (having dose 5+ mSv or unknown dose) or “non-exposed”. Effect modification was tested by adding an interaction term (variable of interest multiplied by the indicator variable) to a model already containing variables for main effects other than radiation exposure. The trend test was carried out using a single variable coded as 1, 2 and 3 for smoking and alcohol drinking. Reported p-values were two-tailed, and a p-value of less than 0.05 was considered statistically significant. Statistical computations were done using the SAS^®^ software (Windows version release 9.1, Cary, the SAS institute).

## RESULTS

Table 1 presents the distribution of cases by demographic variables, histology and atomic-bomb radiation dose. Two thirds were Hiroshima residents, and more than 90% had a histological diagnosis of papillary adenocarcinoma. The mean (range) of age at diagnosis was 43.0 (17-67) years. Only 57 cases (16%) were exposed to atomic bomb radiation, including 18 cases for whom the DS86 estimates were not available.^14^^,^^15^ Duration from diagnosis to interview ranged between 2 and 17 years, with 2 years being the most frequent.

**Table 1.** Characteristics of the thyroid cancer cases.

Item	n	%
City		
Hiroshima	247	68.2
Nagasaki	115	31.8

Sex		
Male	57	15.7
Female	305	84.3

Histology		
Papillary adenocarcinoma	335	92.5
Follicular adenocarcinoma	27	7.5

Age at diagnosis (year)		
<30	31	8.6
30-39	88	24.3
40-49	113	31.2
50-59	92	25.4
60+	38	10.5

Calendar year of diagnosis		
1970-1974	36	9.9
1975-1979	108	29.8
1980-1984	149	41.2
1985-1986	69	19.1

Atomic-bomb exposure		
Born after bombing	77	21.3
Not exposed or negligible dose	228	63.0
Exposed, 5-49 mSv	13	3.6
Exposed, 50-499 mSv	14	3.9
Exposed, 500+ mSv	12	3.3
Exposed, unknown dose	18	5.0

Total	362	100

Because cases and controls were matched pair-wise on city, sex, age, and atomic-bomb exposure, corresponding figures for the controls were exactly the same as for the cases.

Table 2 shows the association between thyroid cancer risk and selected past history and family history of cancer. A high proportion of the cases reported a prior history of goiter or thyroid nodule, resulting in a high OR estimate of 25 for goiter and 5 for nodule, respectively. By contrast, none of hyperthyroidism, hypothyroidism, or other thyroid disease was related to thyroid cancer risk. Five cases had undergone radiotherapy to the head and neck region while none of controls had (Fisher’s exact test, p=0.062). Radiotherapy was given for treatment of goiter (three cases), hyperthyroidism (one case) and thyroid nodule (one case). No association with thyroid cancer was found for history of allergic conditions (asthma, urticaria, etc.), tonsillectomy, or regular use of diuretics or anti-hypertensive agents. Five cases had a family history of thyroid cancer among their parents or siblings (Fisher’s exact test, p=0.062). Having a family history of any cancer was associated with a modest, but significantly increased OR. ORs were especially high for those with a family history of cancer among siblings (2.70) and among sisters (4.25).

**Table 2.** Distribution of thyroid cancer cases and controls and corresponding odds ratios (OR) according to selected past history and family history.

Variable	Cases	Controls	OR (95% CI)*
Disease of the thyroid gland			
Goiter	25	1	25.00 (3.39-184.5)
Nodule	5	1	5.00 (0.58-42.8)
Hyperthyroidism	4	5	0.80 (0.22-2.98)
Hypothyroidism	2	1	2.00 (0.18-22.1)
Other diseases	4	2	2.00 (0.37-10.9)

Radiotherapy to the head and neck region	5	0	infinity

Allergic conditions			
Bronchial asthma	5	4	1.25 (0.34-4.66)
Urticaria	39	40	0.97 (0.60-1.57)
Other allergic diseases	28	22	1.32 (0.73-2.39)
Antihistamines regular use longer than one year	1	2	0.50 (0.05-5.51)

Other medical histories			
Tonsillectomy	5	4	1.25 (0.34-4.66)
Diuretics regular use longer than one year	3	5	0.60 (0.14-2.51)
Anti-hypertensive agents regular use longer than one year	7	16	0.40 (0.16-1.03)

Family history of cancer			
Thyroid cancer, any first-degree relative	5	0	infinity
Any site, any first-degree relative	93	68	1.48 (1.04-2.11)
Any site, parent	70	58	1.24 (0.85-1.79)
Any site, father	48	39	1.27 (0.81-1.98)
Any site, mother	26	23	1.14 (0.64-2.02)
Any site, sibling	28	11	2.70 (1.31-5.58)
Any site, brother	11	6	1.83 (0.68-4.96)
Any site, sister	18	5	4.25 (1.43-12.63)
Any site, son	0	0	-
Any site, daughter	1	1	1.00 (0.06-15.99)

* : Matched on city, age, sex, and radiation exposure

CI: confidence interval

Table 3 shows thyroid cancer risk in association with smoking and alcohol drinking habits. Because smoking and alcohol drinking are closely correlated, the ORs for smoking were adjusted for alcohol consumption and the ORs for drinking were adjusted for smoking, as well as for family history of cancer (any site, any first-degree relative) and past history of thyroid nodules/goiter. The OR for thyroid cancer was significantly decreased for current cigarette smokers compared to those who never smoked. The higher OR for former smokers was not significant. The OR decreased significantly with the increasing number of cigarettes smoked per day. The OR tended to be lower for alcohol drinkers than that for nondrinkers. A dose-related response was suggested when the data were categorized by “never”, “non-daily” and “daily” drinking. These relationships were consistent in the analyses repeated for men and women separately.

**Table 3.** Distribution of thyroid cancer cases and controls and corresponding odds ratios (OR) according to cigarette smoking and alcohol drinking.

Variable		Cases	Controls	Adjusted OR* (95% CI)
Men and women				
Smoking history	Never	292	271	1.00 (reference)
	Past	17	11	1.39 (0.58-3.34)
	Current	53	80	0.46 (0.28-0.76)
	≤15 cig./d	24	30	0.53 (0.27-1.02)
	16+ cig./d	24	43	0.33 (0.16-0.69)
				p trend = 0.001^†^

Alcohol-drinking	Never	172	141	1.00 (reference)
	Non-daily	155	167	0.75 (0.53-1.06)
	Daily	35	54	0.59 (0.35-1.01)
				p trend = 0.032

Men				
Smoking history	Never	14	9	1.00 (reference)
	Past	7	7	0.68 (0.14-3.39)
	Current	36	41	0.57 (0.21-1.56)
	≤15 cig./d	10	9	0.46 (0.10-2.21)
	16+ cig./d	21	28	0.53 (0.18-1.58)
				p trend = 0.28^†^

Alcohol-drinking	Never	11	8	1.00 (reference)
	Non-daily	37	25	1.18 (0.40-3.50)
	Daily	9	24	0.37 (0.10-1.40)
				p trend = 0.032

Women				
Smoking history	Never	278	262	1.00 (reference)
	Past	10	4	2.12 (0.64-6.98)
	Current	17	39	0.38 (0.20-0.71)
	≤15 cig./d	14	21	0.54 (0.26-1.15)
	16+ cig./d	3	15	0.19 (0.06-0.68)
				p trend = 0.002^†^

Alcohol-drinking	Never	161	133	1.00 (reference)
	Non-daily	118	142	0.67 (0.46-0.98)
	Daily	26	30	0.83 (0.44-1.55)
				p trend = 0.14

* : Matched on city, age, sex, and radiation dose; adjusted for family history of cancer and past history of goiter or thyroid nodule; and mutually adjusted for smoking history (never, past, current) and alcohol drinking (never, non-daily, daily).

† : From never” to “current, ≤15 cigarettes/day” and “current, 16+ cigarettes/day”.

CI: confidence interval

We examined the joint effect of smoking and drinking on the risk of thyroid cancer (Table 4). If the effects of smoking and drinking were additive, the OR for those who currently smoke and drink would be expected to be about 1 + (0.73-1) + (0.62-1) = 0.35, and if the effects were multiplicative the OR would be about 0.73 × 0.62 = 0.45. Both of these values were within the 95% CI of 0.14-0.50 for the OR estimate for those who currently smoke and drink. Formal tests for departures from the additive and multiplicative models gave p values of >0.50 and 0.15, respectively. Thus, we could not reject either model.

**Table 4.** Joint effect of smoking and drinking on the risk of thyroid cancer.

Alcohol drinking	Smoking*	

	Never	Current
Never	1.00 (reference)^†^	0.62 (0.27-1.41)
	Ca : Co =146 : 115	Ca : Co = 15 : 15

Current	0.73 (0.51-1.05)	0.26 (0.14-0.50)
	Ca : Co = 141 : 147	Ca : Co = 33 : 58

* : Pairs including a past smoker were deleted from analysis.

† : Odds ratio (95% confidence interval) matched on city, age, sex, and radiation dose, and adjusted for family history of cancer and past history of goiter or thyroid nodule.

Ca : number of cases

Co : number of controls

We repeated all of the above analyses in papillary adenocarcinoma case-control pairs, and found virtually same results (data not shown). Table 5 shows the associations between thyroid cancer and selected non-radiation factors stratified by exposure to atomic-bomb radiation. There was no evidence of effect modification by radiation exposure on ORs associated with history of goiter/thyroid nodule, family history of cancer or cigarette-smoking. The OR associated with alcohol drinking was increased among the radiation-exposed group while it was decreased among the non-exposed, but this difference was of borderline significance (p=0.08).

**Table 5.** Effect modification by radiation-exposure status on the association between non-radiation factors and thyroid cancer risk.

Variable	Non-exposed to radiation^§^			Exposed to radiation^¶^		
	
	Case	Control	OR (95% CI)	Case	Control	OR (95% CI)
History of goiter/nodule*						
No	280	304	1.00 (reference)	52	56	1.00 (reference)
Yes	25	1	25.9 (3.47-192.9)	5	1	3.75 (0.41-34.3)
			*p* for effect modification = 0.20			

Family history of cancer*						
No	235	255	1.00 (reference)	34	39	1.00 (reference)
Yes	70	50	1.55 (1.02-2.37)	23	18	1.43 (0.63-3.26)
			*p* for effect modification = 0.70			

Smoking^†^						
Never	244	226	1.00 (reference)	48	45	1.00 (reference)
Ever	61	79	0.57 (0.34-0.94)	9	12	0.45 (0.12-1.66)
			*p* for effect modification = 0.99			

Alcohol drinking^‡^						
Never	146	111	1.00 (reference)	26	30	1.00 (reference)
Ever	159	194	0.62 (0.43-0.89)	31	27	1.46 (0.62-3.42)
			*p* for effect modification = 0.08			

Matched on city, age, sex, and radiation dose; adjusted for history of goiter or nodule and family history of cancer; and additionally adjusted tor ^†^alcohol drinking (never, ever), ^‡^cigarette smoking (never, ever), or *both.

§ : “Non-exposed” corresponds to “born after bombing” or “not exposed or negligible dose” of “atomic-bomb exposure” in Table 1

¶ : “exposed” corresponds to the other categories in Table 1.

OR: odds ratio

CI : confidence interval

## DISCUSSION

We analyzed medical history, family history, smoking, and alcohol drinking data from an epidemiologic study of thyroid cancer in which cases and controls were enrolled from a well-defined Japanese population. Thyroid cancer cases were ascertained from the population-based tumor registries in Hiroshima and Nagasaki,^10^ and controls were selected from the Life Span Study and F1 cohort populations by matching on demographic variables and on radiation exposure. The reference population is that of the Hiroshima and Nagasaki residents at the time of this study. Pre-structured personal interviews were conducted by trained public health nurses at the time of this study. Despite the low incidence of thyroid cancer,^1^^,^^2^ the present study collected a fairly large number of cases, all of which were histologically confirmed by pathologists. In addition, the response rate was high and nearly identical for the cases (88%) and controls (87%). Although some of the data from this study were included in international pooled analyses,^4^^,^^6^ the present analysis addressed specific questions not considered in the pooled analyses. We analyzed past medical history of non-thyroidal diseases; family history of cancer; smoking and alcohol drinking considering dose-response relationship, mutual confounding effects, and interaction between the factors. We also examined possible effect modification by radiation exposure, which is especially relevant for the population of Hiroshima and Nagasaki, although the small number (n=39) of case-control pairs for whom dose estimate was available did not allow a detailed analysis as previously done for breast cancer.^16^

The cases identified from the local population tumor registries included persons who had moved into Hiroshima or Nagasaki after the Life Span Study or F1 cohort was established decades before the present study while all controls were from one of the above cohorts. A potential selection bias may have occurred if thyroid cancer patients with certain characteristics, e.g., a history of goiter, a habit of smoking, etc., selectively moved to Hiroshima or Nagasaki. To assess the possible impact of such bias, we repeated analyses with 86 cases who were the members of the Life Span Study or F1 cohort and their counterpart controls. The results generally duplicated the findings based on all subjects. The point estimates of OR were 8.00 for history of goiter or thyroid nodule, 1.83 for history of any cancer in siblings, 2.00 for history of any cancer in sisters, 0.45 for current vs. never smokers, and 0.93 for daily vs. never drinkers, respectively.

Some of the thyroid cancer patients were prevalent cases, including those who had survived up to 17 years after diagnosis. Since patients with thyroid cancer, especially papillary adenocarcinoma or follicular adenocarcinoma, have excellent survival experience,^17^ use of prevalent cases would not be a significant source of selection bias, because attrition due to death, which would have been largely from causes other than thyroid cancer, is likely to have occurred almost equally among those with and without thyroid cancer. However, because smoking and alcohol drinking are generally considered “unhealthy”, some cases may have quit these habits or reduced the amount and frequency of cigarette smoking or alcohol consumption after cancer diagnosis. The subjects in this study were asked about their habits before diagnosis (or index time), but it may have been difficult for them to recall the exact date of diagnosis for often dormant papillary/follicular carcinoma of the thyroid. We thus repeated the analysis of smoking and drinking with subjects to 182 case-control pairs in which the interview was carried out within 5 years after the index time. The results were similar to those based on all subjects. The OR for current smokers relative to nonsmokers adjusted for drinking was 0.41 (95% CI: 0.20-0.84), and the OR for daily drinkers relative to nondrinkers adjusted for smoking was 0.31 (95% CI: 0.13-0.72), adjusted as well for family history of cancer and past history of goiter/nodule.

The inverse association of thyroid cancer with smoking and alcohol drinking observed in the present study might have been confounded by factors that the present study could not consider. People with a high socioeconomic status or who highly care about their health are less likely to be a smoker or a heavy drinker, and also would be more sensitively aware of their bodily changes or more ready to visit a clinic, leading to a higher chance of thyroid cancer diagnosis. Such potential confounding bias associated with socioeconomic status or health consciousness may not be small for papillary adenocarcinoma of the thyroid gland, which is of a highly dormant and silent nature as indicated by a high prevalence rate (6-36 %) of latent papillary carcinoma at autopsy series.^18^

Although not all,^19^^-^^22^ several previous studies of thyroid cancer have reported a reduced risk of thyroid cancer significantly,^23^^-^^26^ or not significantly associated with cigarette smoking.^27^^-^^33^ Although results are less consistent,^20^^,^^22^^,^^24^^,^^29^^,^^31^^-^^33^ several studies have also suggested reduced thyroid cancer risk associated with alcohol drinking.^19^^,^^25^^,^^27^^,^^31^^,^^34^ A number of possible mechanisms have been suggested. Smoking may reduce thyroid-stimulating hormone (TSH) levels, which can promote thyroid hyperplasia leading to thyroid cancer.^35^^,^^36^ Lower TSH levels in smokers than in former and never-smokers have been reported by some,^37^^-^^40^ but not all studies.^41^ Another proposed biological pathway is the anti-estrogenic effect of smoking.^42^ Estrogenic activity is thought to promote thyroid carcinogenesis. Although estrogen levels do not seem to be different between smokers and non-smokers,^43^^-^^45^ smoking may decrease the bioavailability of estrogens at target tissues.^6^^,^^46^^-^^48^ Radiation exposure is considered to induce initial damage while hormonal changes due to smoking may be involved in a later stage of carcinogenesis, suggesting a possible multiplicative interaction. There was, however, no indication in the present data of a multiplicative interaction between smoking and radiation.

In the international pooled analysis, adjustment for current smoking eliminated an apparent protective effect of alcohol drinking.^6^ Rossing et al, however, reported a decreased risk of papillary thyroid cancer in women associated with alcohol consumption after controlling for smoking.^34^ In the present study, we found the significant effects of drinking after adjustment for smoking, and of smoking after adjustment for drinking. However, the findings are based on the relatively small number of subjects, and on crude measures of the intensity of alcohol consumption.

A strong association of thyroid cancer with a history of goiter or benign nodules has previously been reported.^19^^,^^24^^,^^27^^,^^29^^,^^30^^,^^49^^-^^51^ Possible potential bias in reporting of previous thyroid disease by cases cannot be ruled out, but seems unlikely because the significantly elevated risk was specifically related to goiter or thyroid nodule but not to other thyroid diseases. Our OR estimated for goiter or nodule of 15.0 (95% CI: 3.6-62.7) is consistent with the estimates of 5.9 for a history of goiter and 29.9 for a history of benign nodules/adenomas as reported by Franceschi et al from the international pooled analysis which included our data.^4^ History of thyroid goiter and nodule is one of the strongest risk factors for thyroid cancer apart from radiation in childhood, although it remains unclear whether thyroid nodules are cancer precursor lesions or share a common etiologic background with thyroid cancer.

While several studies showed an increased risk of thyroid cancer linked to a family history of this cancer, the nature of the relationship between thyroid cancer and a family history of other cancers is not clear.^52^ We found five cases with a family history of thyroid cancer compared to none of the controls, but the difference was of borderline significance. We found a significantly increased OR associated with a cancer history in siblings, especially in sisters. The apparent relationships with family cancer history need to be interpreted carefully and potential recall bias in cancer cases must be considered. The generally high ORs associated with a history of cancer in any members of the family, including first-degree relatives, in our study tend to support the possibility of a potential recall bias by thyroid cancer patients.

Studies of the atomic bomb survivors and other irradiated populations have demonstrated a high risk of thyroid cancer associated with exposure in childhood and adolescence.^1^^,^^3^ In the present study, the proportion and number of cases exposed to the atomic bombs at age 20 years or younger were small. Therefore, the lack of a significant interaction between radiation exposure and nonradiation factors, such as history of goiter/nodule, family history of cancer, smoking, and alcohol drinking, may be due to insufficient statistical power.

Recently attention has been given to an increasing trend of thyroid cancer in the United States^53^ and other countries.^54^^,^^55^ Increasing trend of thyroid cancer was also observed previously in Japan,^56^ although updated analysis of thyroid cancer trends is desirable. The increasing trend may in part be attributed to improved diagnosis or detection but also reflect changes in risk factors. The latest worldwide cancer incidence data also indicate that thyroid cancer rates are high in Japan as well as in Australia, New Zealand, and Central America.^2^ Studies of risk factors in populations with high thyroid cancer rates may provide useful insights into possible risk determinants. The present analysis focused on medical history, family history, smoking and alcohol drinking in the case-control study to examine a variety of possible risk factors. Results of analyses of other factors will be forthcoming. The present paper showed that history of goiter or thyroid nodule is a significant risk factor for thyroid cancer in Japan as in other countries. The apparent association with family history of thyroid and other cancer suggests host susceptibility, either genetic or otherwise, but is uncertain due to the limitations with self-reported retrospective information, and needs corroboration by others using different methods. Cigarette smoking was found to reduce thyroid cancer risk and alcohol drinking may also have a protective effect independent of smoking. The inverse relationship between these two factors and thyroid cancer is strange in view of the finding that smokers have increased risk of other thyroid pathologies, such as goiter and nodules,^57^^,^^58^ which predispose individuals with these conditions to an increased risk of thyroid cancer. In addition to learning more about the hormonal and other biological effects of smoking and drinking, further epidemiologic research is needed to clarify the nature of the inverse association with smoking and drinking, as prospective data available to date^20^^,^^22^ have failed to confirm this association. Thyroid cancer is relatively infrequent and less fatal than most other malignancies, but presents a significant morbidity burden in the community and warrants more epidemiologic attention than previously given.

## References

[1] Ron E. Epidemiology of thyroid cancer. In: Schottenfeld D, Fraumeni JJ, eds. Cancer Epidemiology and Prevention. Oxford University Press. Oxford, 1996: 1000-21.

[2] Parkin D, Whelan S, Ferlay J, Teppo L, Thomas D. Cancer Incidence in Five Continents, Vol. VIII. International Agency for Research on Cancer. Lyon, 2003.

[3] Thompson DE, Mabuchi K, Ron E, Soda M, Tokunaga M, Ochikubo S, Cancer incidence in atomic bomb survivors. Part II: Solid tumors, 1958-1987. Radiat Res 1994;137:S17-67. 8127952

[4] Franceschi S, Preston-Martin S, Dal Maso L, Negri E, La Vecchia C, Mack WJ, A pooled analysis of case-control studies of thyroid cancer. IV. Benign thyroid diseases. Cancer Causes Control 1999;10:583-95. 10616827 10.1023/a:1008907227706

[5] World Cancer Research Fund, American Institute for Cancer Research, eds. Food, nutrition and the prevention of cancer: a global perspective. World Cancer Research Fund, American Institute for Cancer Research. Wahington, DC, 1997. 10.1016/s0899-9007(99)00021-0 10378216

[6] Mack WJ, Preston-Martin S, Dal Maso L, Galanti R, Xiang M, Franceschi S, A pooled analysis of case-control studies of thyroid cancer: cigarette smoking and consumption of alcohol, coffee, and tea. Cancer Causes Control 2003;14:773-85. 14674742 10.1023/a:1026349702909

[7] Ezaki H, Ebihara S, Fujimoto Y, Iida F, Ito K, Kuma K, Analysis of thyroid carcinoma based on material registered in Japan during 1977-1986 with special reference to predominance of papillary type. Cancer 1992;70:808-14. 1643612 10.1002/1097-0142(19920815)70:4<808::aid-cncr2820700415>3.0.co;2-l

[8] Takezaki T, Hirose K, Inoue M, Hamajima N, Kuroishi T, Nakamura S, Risk factors of thyroid cancer among women in Tokai, Japan. J Epidemiol 1996;6:140-7. 8952218 10.2188/jea.6.140

[9] Negri E, Ron E, Franceschi S, Dal Maso L, Mark SD, Preston-Martin S, A pooled analysis of case-control studies of thyroid cancer. I. Methods. Cancer Causes Control 1999;10:131-42. 10231162 10.1023/a:1008851613024

[10] Mabuchi K, Soda M, Ron E, Tokunaga M, Ochikubo S, Sugimoto S, Cancer incidence in atomic bomb survivors. Part 1: Use of the tumor registries in Hiroshima and Nagasaki for incidence studies. Radiat Res 1994;137:S1-16. 8127951

[11] Preston DL, Shimizu Y, Pierce DA, Suyama A, Mabuchi K Studies of mortality of atomic bomb survivors. Report 13: Solid cancer and noncancer disease mortality: 1950-1997. Radiat Res 2003;160:381-407. 12968934 10.1667/rr3049

[12] Izumi S, Suyama A, Koyama K Radiation-related mortality among offspring of atomic bomb survivors: a half-century of follow-up. Int J Cancer 2003;107:292-7. 12949810 10.1002/ijc.11400

[13] Yoshimoto Y, Schull W, Kato H, Neel JV Mortality among the offspring (F1) of atomic bomb survivors, 1946-85. J Radiat Res 1991;32:327-51. 10.1269/jrr.32.327 1817186

[14] Roesh WC. Final report on the reassessment of atomic bomb radiation dosimetry in Hiroshima and Nagasaki. RERF. Hiroshima, 1987.

[15] Pierce DA, Shimizu Y, Preston DL, Vaeth M, Mabuchi K Studies of the mortality of atomic bomb survivors. Report 12, Part I. Cancer: 1950-1990. Radiat Res 1996;146:1-27. 8677290

[16] Land CE, Hayakawa N, Machado SG, Yamada Y, Pike MC, Akiba S, A case-control interview study of breast cancer among Japanese A-bomb survivors. II. Interactions with radiation dose. Cancer Causes Control 1994;5:167-76. 8167264 10.1007/BF01830263

[17] Hiroshimaya T, Takahashi H, Yao K, Inagi K, Nakayama M, Makoshi T, Clinical review of thyroid malignant tumor. Acta Oto-Laryngol Suppl 2002:85-7. 10.1080/000164802760057653 12212603

[18] Ottino A, Pianzola HM, Castelletto RH Occult papillary thyroid carcinoma at autopsy in La Plata, Argentina. Cancer 1989;64:547-51. 2736500 10.1002/1097-0142(19890715)64:2<547::aid-cncr2820640232>3.0.co;2-n

[19] Preston-Martin S, Bernstein L, Pike MC, Maldonado AA, Henderson BE Thyroid cancer among young women related to prior thyroid disease and pregnancy history. Br J Cancer 1987;55:191-5. 3814488 10.1038/bjc.1987.36 PMC2002082

[20] Iribarren C, Haselkorn T, Tekawa IS, Friedman GD Cohort study of thyroid cancer in a San Francisco Bay area population. Int J Cancer 2001;93:745-50. 11477590 10.1002/ijc.1377

[21] Mack WJ, Preston-Martin S, Bernstein L, Qian D Lifestyle and other risk factors for thyroid cancer in Los Angeles County females. Ann Epidemiol 2002;12:395-401. 12160598 10.1016/s1047-2797(01)00281-2

[22] Navarro Silvera SA, Miller AB, Rohan TE Risk factors for thyroid cancer: a prospective cohort study. Int J Cancer 2005;116:433-8. 15818623 10.1002/ijc.21079

[23] Hallquist A, Hardell L, Degerman A, Boquist L Occupational exposures and thyroid cancer: results of a case-control study. Eur J Cancer Prev 1993;2:345-9. 8358287 10.1097/00008469-199307000-00009

[24] D’Avanzo B, La Vecchia C, Franceschi S, Negri E, Talamini R History of thyroid diseases and subsequent thyroid cancer risk. Cancer Epidemiol Biomarkers Prev 1995;4:193-9. 7606193

[25] Linos A, Linos DA, Vgotza N, Souvatzoglou A, Koutras DA Does coffee consumption protect against thyroid disease? Acta Chir Scand 1989;155:317-20. 2816215

[26] Kreiger N, Parkes R Cigarette smoking and the risk of thyroid cancer. Eur J Cancer 2000;36:1969-73. 11000579 10.1016/s0959-8049(00)00198-2

[27] Kolonel LN, Hankin JH, Wilkens LR, Fukunaga FH, Hinds MW An epidemiologic study of thyroid cancer in Hawaii. Cancer Causes Control 1990;1:223-34. 2102295 10.1007/BF00117474

[28] McTiernan AM, Weiss NS, Daling JR Incidence of thyroid cancer in women in relation to reproductive and hormonal factors. Am J Epidemiol 1984;120:423-35. 6475918 10.1093/oxfordjournals.aje.a113907

[29] Ron E, Kleinerman RA, Boice JD Jr, LiVolsi VA, Flannery JT, Fraumeni JF Jr A population-based case-control study of thyroid cancer. J Natl Cancer Inst 1987;79:1-12. 3474436

[30] Wingren G, Hatschek T, Axelson O Determinants of papillary cancer of the thyroid. Am J Epidemiol 1993;138:482-91. 8213752 10.1093/oxfordjournals.aje.a116882

[31] Galanti MR, Hansson L, Bergstrom R, Wolk A, Hjartaker A, Lund E, Diet and the risk of papillary and follicular thyroid carcinoma: a population-based case-control study in Sweden and Norway. Cancer Causes Control 1997;8:205-14. 9134245 10.1023/a:1018424430711

[32] Glattre E, Haldorsen T, Berg JP, Stensvold I, Solvoll K Norwegian case-control study testing the hypothesis that seafood increases the risk of thyroid cancer. Cancer Causes Control 1993;4:11-6. 8431525 10.1007/BF00051708

[33] Levi F, Franceschi S, Gulie C, Negri E, La Vecchia C Female thyroid cancer: the role of reproductive and hormonal factors in Switzerland. Oncology 1993;50:309-15. 8497382 10.1159/000227201

[34] Rossing MA, Cushing KL, Voigt LF, Wicklund KG, Daling JR Risk of papillary thyroid cancer in women in relation to smoking and alcohol consumption. Epidemiology 2000;11:49-54. 10615843 10.1097/00001648-200001000-00011

[35] Henderson BE, Ross RK, Pike MC, Casagrande JT Endogenous hormones as a major factor in human cancer. Cancer Res 1982;42:3232-9. 7046921

[36] Williams ED TSH and thyroid cancer. Horm Metab Res 1990;23:72-5. 2210635

[37] Eden S, Jagenburg R, Lindstedt G, Lundberg PA, Mellstrom D Thyroregulatory changes associated with smoking in 70-year-old men. Clin Endocrinol (Oxf) 1984;21:605-10. 6439438 10.1111/j.1365-2265.1984.tb01402.x

[38] Christensen SB, Ericsson UB, Janzon L, Tibblin S, Melander A Influence of cigarette smoking on goiter formation, thyroglobulin, and thyroid hormone levels in women. J Clin Endocrinol Metab 1984;58:615-8. 6699129 10.1210/jcem-58-4-615

[39] Ericsson UB, Lindgarde F Effects of cigarette smoking on thyroid function and the prevalence of goitre, thyrotoxicosis and autoimmune thyroiditis. J Intern Med 1991;229:67-71. 1995765 10.1111/j.1365-2796.1991.tb00308.x

[40] Fisher CL, Mannino DM, Herman WH, Frumkin H Cigarette smoking and thyroid hormone levels in males. Int J Epidemiol 1997;26:972-7. 9363517 10.1093/ije/26.5.972

[41] Karakaya A, Tuncel N, Alptuna G, Kocer Z, Erbay G Influence of cigarette smoking on thyroid hormone levels. Hum Toxicol 1987;6:507-9. 3692496 10.1177/096032718700600610

[42] Baron JA, La Vecchia C, Levi F The antiestrogenic effect of cigarette smoking in women. Am J Obstet Gynecol 1990;162:502-14. 2178432 10.1016/0002-9378(90)90420-c

[43] Berta L, Fortunati N, Gennari P, Appendino M, Casella A, Frairia R Influence of cigarette smoking on pituitary and sex hormone balance in healthy premenopausal women. Fertil Steril 1991;56:788-9. 1915961 10.1016/s0015-0282(16)54620-2

[44] Cassidenti DL, Pike MC, Vijod AG, Stanczyk FZ, Lobo RA A reevaluation of estrogen status in postmenopausal women who smoke. Am J Obstet Gynecol 1992;166:1444-8. 1534446 10.1016/0002-9378(92)91617-j

[45] Thomas EJ, Edridge W, Weddell A, McGill A, McGarrigle HH The impact of cigarette smoking on the plasma concentrations of gonadotrophins, ovarian steroids and androgens and upon the metabolism of oestrogens in the human female. Hum Reprod 1993;8:1187-93. 8408515 10.1093/oxfordjournals.humrep.a138226

[46] Cassidenti DL, Vijod AG, Vijod MA, Stanczyk FZ, Lobo RA Short-term effects of smoking on the pharmacokinetic profiles of micronized estradiol in postmenopausal women. Am J Obstet Gynecol 1990;163:1953-60. 2256508 10.1016/0002-9378(90)90780-b

[47] Michnovicz JJ, Hershcopf RJ, Naganuma H, Bradlow HL, Fishman J Increased 2-hydroxylation of estradiol as a possible mechanism for the anti-estrogenic effect of cigarette smoking. N Engl J Med 1986;315:1305-9. 3773953 10.1056/NEJM198611203152101

[48] Michnovicz JJ, Hershcopf RJ, Haley NJ, Bradlow HL, Fishman J Cigarette smoking alters hepatic estrogen metabolism in men: implications for atherosclerosis. Metabolism 1989;38:537-41. 2725292 10.1016/0026-0495(89)90213-8

[49] McTiernan AM, Weiss NS, Daling JR Incidence of thyroid cancer in women in relation to previous exposure to radiation therapy and history of thyroid disease. J Natl Cancer Inst 1984;73:575-81. 6590909

[50] Preston-Martin S, Jin F, Duda MJ, Mack WJ A case-control study of thyroid cancer in women under age 55 in Shanghai (People’s Republic of China). Cancer Causes Control 1993;4:431-40. 8218875 10.1007/BF00050862

[51] Levi F, Franceschi S, La Vecchia C, Negri E, Gulie C, Duruz G, Previous thyroid disease and risk of thyroid cancer in Switzerland. Eur J Cancer 1991;27:85-8. 1826448 10.1016/0277-5379(91)90069-p

[52] Memon A, Berrington De Gonzalez A, Luqmani Y, Suresh A Family history of benign thyroid disease and cancer and risk of thyroid cancer. Eur J Cancer 2004;40:754-60. 15010077 10.1016/j.ejca.2003.12.011

[53] Davies L, Welch HG Increasing incidence of thyroid cancer in the United States, 1973-2002. JAMA 2006;295:2164-7. 16684987 10.1001/jama.295.18.2164

[54] Reynolds RM, Weir J, Stockton DL, Brewster DH, Sandeep TC, Strachan MW Changing trends in incidence and mortality of thyroid cancer in Scotland. Clin Endocrinol (Oxf) 2005;62:156-62. 15670190 10.1111/j.1365-2265.2004.02187.x

[55] Smailyte G, Miseikyte-Kaubriene E, Kurtinaitis J Increasing thyroid cancer incidence in Lithuania in 1978-2003. BMC Cancer 2006;6:284. 17156468 10.1186/1471-2407-6-284 PMC1764427

[56] Koike A, Naruse T Incidence of thyroid cancer in Japan. Semin Surg Oncol 1991;7:107-11. 2034935 10.1002/ssu.2980070211

[57] Galanti MR, Granath F, Cnattingius S, Ekbom-Schnell A, Ekbom A Cigarette smoking and the risk of goitre and thyroid nodules amongst parous women. J Intern Med 2005;258:257-64. 16115300 10.1111/j.1365-2796.2005.01523.x

[58] Vestergaard P Smoking and thyroid disorders--a meta-analysis. Eur J Endocrinol 2002;146:153-61. 11834423 10.1530/eje.0.1460153

